# Crystallographic home-source X-ray data for the atomic-resolution experimental phasing of the Shank3 SH3 domain structure from pseudomerohedrally twinned crystals

**DOI:** 10.1016/j.dib.2018.09.040

**Published:** 2018-09-18

**Authors:** Srinivas Kumar Ponna, Matti Myllykoski, Petri Kursula

**Affiliations:** a Faculty of Biochemistry and Molecular Medicine & Biocenter Oulu, University of Oulu, Finland; b Department of Biomedicine, University of Bergen, Norway

**Keywords:** Protein crystallography, Diffraction, Experimental phasing, Data collection, Home-source X-ray diffractometer

## Abstract

By far most macromolecular crystallographic data collection and experimental phasing is nowadays carried out using synchrotron radiation. Here, we present two crystallographic datasets collected on a home-source X-ray diffractometer, which can *per se* be use to experimentally solve the atomic-resolution crystal structure of the Src homology 3(SH3)-like domain from the postsynaptic protein Shank3. The refined structure was described in the article “Structure of an unconventional SH3 domain from the postsynaptic density protein Shank3 at ultrahigh resolution” (Ponna et al., 2017) [1]. Crystals of the Shank3 SH3 domain were derivatized through soaking in 1 M sodium iodide prior to diffraction data collection at a wavelength of 1.54 Å. High-resolution data are reported for a native crystal to 1.01 Å and an iodide-derivatized one to 1.60 Å. The crystals suffered from several anomalies affecting experimental phasing: a high fraction (34–40%) of pseudomerohedral twinning, significant pseudotranslational symmetry (> 15%) with the operator 0.5,0,0.5, and a low solvent content. Twinning with the operator *h,-k,-l* is made possible by the space group *P*2_1_ coupled with a unit cell *β* angle of 90.0°. The data can be used to repeat and optimize derivatization and phasing procedures, to understand halide interactions with protein surfaces, to promote the use of home X-ray sources for protein structure determination, as well as for educational purposes and protocol development.

**Specifications table**

**Table:** 

Subject area	*Biology*
More specific subject area	*Structural biology, X-ray crystallography*
Type of data	*X-ray diffraction datasets, graphs, tables*
How data was acquired	*Home-source X-ray diffraction data collection at a wavelength of 1.54 Å at 100 K. Data collected using the rotation method were reduced and scaled for structure determination. Data were similarly collected for a native crystal and a crystal soaked in an iodide solution.*
Data format	*Processed data from diffraction images in ASCII format*
Experimental factors	*Data from protein crystals subjected to heavy atom derivatization*
Experimental features	*Crystals of the Shank3 SH3 domain were derivatized with iodide, and anomalous and native diffraction datasets were collected using home-source X-rays.*
Data source location	*Biocenter Oulu X-ray Crystallography Core Facility, Oulu, Finland*
Data accessibility	*The derivative and native data are presented in this article and available as supplementary material.*
Related research article	[1] S.K. Ponna, M. Myllykoski, T.M. Boeckers, P. Kursula, Structure of an unconventional SH3 domain from the postsynaptic density protein Shank3 at ultrahigh resolution., Biochem Biophys. Res. Commun. 490 (2017) 806–812

**Value of the data**

● Atomic-resolution structure solution for the Shank3 SH3 domain, based on home-source data, can be reproduced.
● Iodide ion binding to proteins can be understood.
● Derivatization and data collection conditions for protein crystals at the home source can be optimized.
● Various experimental phasing approaches can be employed, such as SAD (single-wavelength anomalous dispersion) and SIRAS (single isomorphous replacement with anomalous signal).
● The effects of twinning, pseudotranslational symmetry, and low solvent content on experimental phasing can be assessed.

## 1 Data

**Fig. 1:**
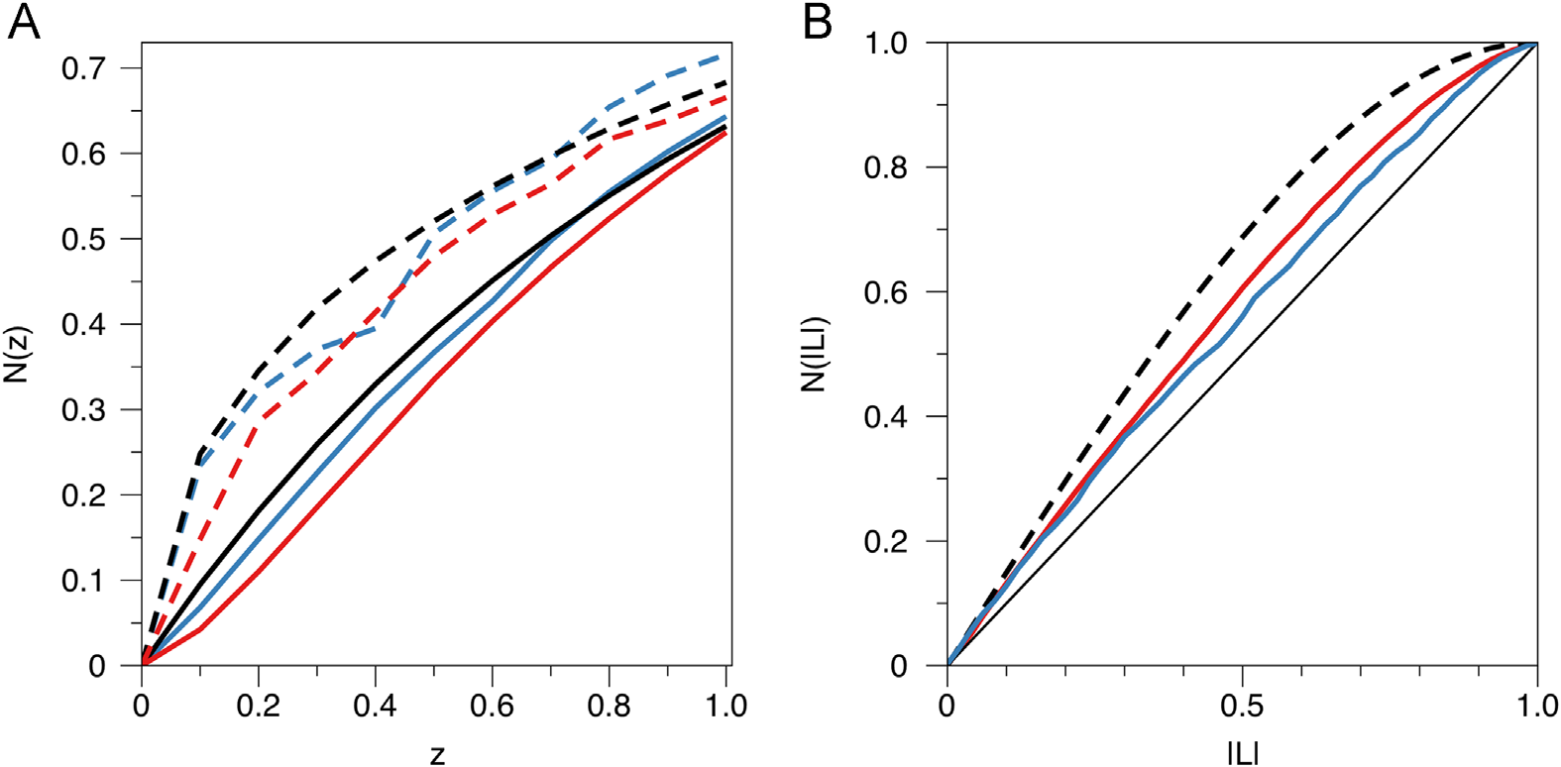
Tests for twinning affected by pseudotranslational symmetry. A) The cumulative intensity distribution indicates no twinning for the derivative dataset. The dashed lines represent centric reflections and the solid lines acentric reflections. Black, theoretical; red, native data; blue, derivative data. Note how especially the derivative data shows very little signs of twinning. B) The *L*-test [6] for acentric reflections indicates similar and significant pseudomerohedral twinning fractions for both the native and the iodide-derivatized dataset. Solid black line, theoretical nontwinned; dashed black line, theoretical perfectly (50%) twinned; red, native data; blue, derivative data. It is clear that both datasets have a high degree of twinning, and the fraction is similar in both datasets.

**Fig. 2:**
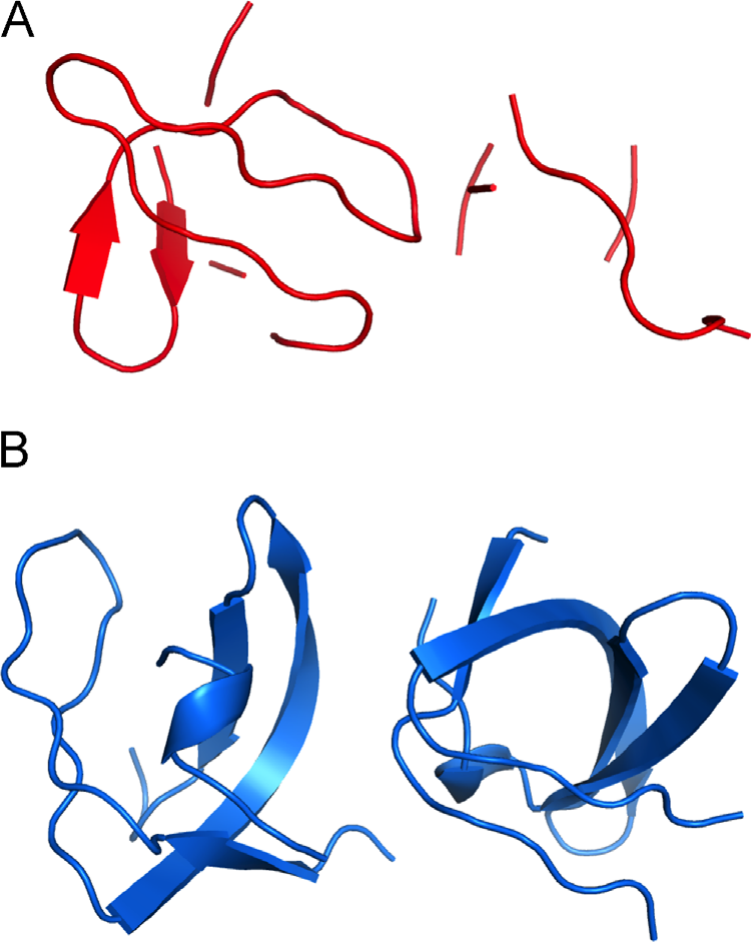
Results that can be typically obtained from automated workflows using the presented data**.** Structures automatically built with A) SAD and B) SIRAS approaches, in the AutoRickshaw pipeline [7]. While the SIRAS method yields a near-complete model with two monomers in the asymmetric unit, the SAD protocol, using only the derivative data, also builds one of the monomers well enough to finalize the structure.

We recently solved the crystal structure of the unconventional SH3 domain from Shank3 [1], and the high-resolution native data [2] collected using a synchrotron source were deposited at the Protein Data Bank. However, originally the basis for structure solution was diffraction data collected on the home laboratory X-ray source for both a native and a derivatized crystal. We provide the original home-source data used for solving the Shank3 SH3 domain structure at atomic resolution. The crystallographic data are deposited in Supplementary Material, in the format of the SCALEPACK data processing and scaling program [3]; this text-based format can easily be converted to those used by mainstream crystallography software, or it can be used directly by most programs. Twinning and pseudotranslation properties in the datasets are presented in Table 1, Fig. 1 shows the detection of twinning in the datasets with different protocols, and Fig. 2 shows typical results from automatic structure solution workflows with SIRAS and SAD protocols.

**Table 1:** Properties of the collected datasets. Processing statistics have been presented elsewhere [1].

Dataset	Native	Iodide derivative
Space group	*P*2_1_	*P*2_1_
Unit cell	*a* = 29.615 Å, *b* = 52.616 Å, *c* = 31.55Å, *β* = 90.00°	*a* = 29.632 Å, *b* = 52.634 Å, *c* = 31.593 Å, *β* = 90.047°
Resolution range (Å)	30–1.01	52–1.62
Twinning operator	*h*,-*k*,-*l*	*h*,-*k*,-*l*
Twinning fraction (%) (*H*-test [8]/Britton test [9])	34.7/34.9	40.0/34.5
Pseudotranslation operator	0.5,0,0.5	0.5,0,0.5
Pseudotranslation fraction (%)	15.8	21.2

A low solvent content in general is adversary to phasing, as bulk solvent correction becomes ineffective. In the case of Shank3-SH3 crystals, the solvent content can be estimated at 32%, not taking into account ordered solvent around the protein. Hence, actual bulk solvent in these crystals is at a very low level. In addition, twinning and pseudotranslation in the crystal can make experimental phasing very difficult. In the case of the Shank3 SH3 domain, the crystal form is unfortunate in having *P*2_1_ symmetry, with a *β* angle of 90.0°, enabling high degrees of pseudomerohedral symmetry through the operator *h,-k,-l*. The datasets used for phasing and described here have twinning fractions ~35% (Table 1), and the highest-resolution synchrotron dataset – eventually not used in refinement – was nearly perfectly twinned [1]. The crystals also suffer from moderate pseudotranslational symmetry, in the order of ~ 15–20%, which counteracts twinning analyses and affects statistics (Fig. 1, Table 1). Especially for the derivative dataset, traditional cumulative intensity statistics appear normal, while the *L* test clearly indicates a similar level of twinning as for the native dataset (Fig. 1).

The Shank3-SH3 structure was originally solved using a SIRAS approach, utilizing both the native and derivatized datasets [1], [2]. The structure can also be solved using the iodide-derivatized crystal alone, although refining and rebuilding the structure in this case will require more effort (Fig. 2). Due to the high resolution of the native data, automated SIRAS procedures, such as that implemented in the AutoRickshaw workflow [9], produce a nearly complete, atomic-resolution model of the Shank3 SH3 domain using the home-source data described here, without any user intervention.

## 2 Experimental design, materials, and methods

The preparation of recombinant protein and crystallization for the Shank3 SH3 domain have been described [2]. The derivatization by soaking in sodium iodide has been published [1]. Data collection was performed at the Biocenter Oulu X-ray crystallography core facility at the fixed Cu-Kα wavelength of 1.54 Å. Data collection temperature was 100 K, under a stream of gaseous nitrogen. Data were processed using SAINT and SADABS (Bruker).

Analysis of the data quality for phasing was done using SHELXC [4], and Xtriage [5] was used to analyze dataset properties, including twinning and pseudotranslation. The final refined crystal structure has been published elsewhere [1] and deposited at the PDB with the entry code 5o99, together with the 0.87-Å dataset collected using synchrotron radiation [2] and used for final refinement [1].

## Supplementary files

The supplementary archive contains the two crystallographic datasets, corresponding to the data discussed above and shown in Table 1 and Fig. 1. The data are in the ASCII format of the software SCALEPACK [3], and named as follows: native.sca, iodide.sca
